# Toosendanin targeting eEF2 impedes Topoisomerase I & II protein translation to suppress esophageal squamous cell carcinoma growth

**DOI:** 10.1186/s13046-023-02666-5

**Published:** 2023-04-24

**Authors:** Xuechao Jia, Penglei Wang, Chuntian Huang, Dengyun Zhao, Qiong wu, Bingbing Lu, Wenna Nie, Limeng Huang, Xueli Tian, Pan li, Kyle Vaughn Laster, Yanan Jiang, Xiang Li, Honglin Li, Zigang Dong, Kangdong Liu

**Affiliations:** 1 Department of Pathophysiology, School of Basic Medical Sciences, Academy of Medical Sciences, Zhengzhou University, Zhengzhou, 450000 Henan China; 2 China-US (Henan) Hormel Cancer Institute, Zhengzhou, 450000 Henan China; 3 Department of Pathology and Pathophysiology, Henan University of Traditional Chinese Medicine, Zhengzhou, 450000 Henan China; 4 Innovation Center for AI and Drug Discovery, East China Normal University, Shanghai, 200062 China; 5 Lingang Laboratory, Shanghai, 200031 China; 6 Basic Medicine Sciences Research Center, Academy of Medical Sciences, Zhengzhou University, Zhengzhou, 450052 Henan China; 7 State Key Laboratory of Esophageal Cancer Prevention and Treatment, Zhengzhou University, Zhengzhou, 450000 Henan China; 8 The Collaborative Innovation Center of Henan Province for Cancer Chemoprevention, Zhengzhou, 450000 Henan China; 9 Provincial Cooperative Innovation Center for Cancer Chemoprevention, Zhengzhou University, Zhengzhou, 450000 Henan China; 10 Tianjian Advanced Biomedical Laboratory, Zhengzhou, 450052 Henan China

**Keywords:** ESCC, eEF2, RIP-Sequence, ITC, Toosendanin

## Abstract

**Background:**

Although molecular targets such as HER2, TP53 and PIK3CA have been widely studied in esophageal cancer, few of them were successfully applied for clinical treatment. Therefore, it is urgent to discover novel actionable targets and inhibitors. Eukaryotic translational elongation factor 2 (eEF2) is reported to be highly expressed in various cancers. However, its contribution to the maintenance and progression of cancer has not been fully clarified.

**Methods:**

In the present study, we utilized tissue array to evaluate eEF2 protein expression and clinical significance in esophageal squamous cell carcinoma (ESCC). Next, we performed knockdown, overexpression, RNA-binding protein immunoprecipitation (RIP) sequence, and nascent protein synthesis assays to explore the molecular function of eEF2. Furthermore, we utilized compound screening, Surface Plasmon Resonance (SPR), Isothermal Titration Calorimetry (ITC) assay, cell proliferation and Patient derived xenograft (PDX) mouse model assays to discover an eEF2 inhibitor and assess its effects on ESCC growth.

**Results:**

We found that eEF2 were highly expressed in ESCC and negatively associated with the prognosis of ESCC patients. Knocking down of eEF2 suppressed the cell proliferation and colony formation of ESCC. eEF2 bond with the mRNA of Topoisomerase II (TOP1) and Topoisomerase II (TOP2) and enhanced the protein biosynthesis of TOP1 and TOP2. We also identified Toosendanin was a novel inhibitor of eEF2 and Toosendanin inhibited the growth of ESCC in vitro and in vivo.

**Conclusions:**

Our findings show that Toosendanin treatment suppresses ESCC growth through targeting eEF2 and regulating downstream TOP1 and TOP2 biosynthesis. eEF2 could be supplied as a potential therapeutic target in the further clinical studies.

**Supplementary Information:**

The online version contains supplementary material available at 10.1186/s13046-023-02666-5.

## Background

Multi-disciplinary approaches have been widely utilized to improve the clinical treatment of esophageal squamous cell carcinoma (ESCC); however, the prognosis of ESCC is still poor [1]. The identification of novel biomarkers capable of accurately predicting treatment response and prognosis is a valuable strategy for increasing patient survival and quality of life [2, 3]. Compromised signaling pathways in ESCC can be broadly grouped into five categories: receptor tyrosine kinases (such as HER2, EGFR, PIK3CA), cell cycle regulators (such as TP53, CDKN2A), proliferation and differentiation (TP63, SOX2), chromatin remodeling (KMD6A, KMT2D) and immune checkpoints (such as PD1, PD-L1). Of the aforementioned pathways, all inhibitors except those targeting PD1 and PD-L1 have failed to produce favorable clinical responses in patients. However, the median overall survival time of PD1/PD-L1 positive patients treated with inhibitors increased by approximately 3 months [4–6]. Thus, it is imperative to discover novel actionable targets and its inhibitors.

In most cancers, aberrant regulation of the translation machinery results in the abnormal synthesis of molecules that could potentially enhance cell proliferation [7, 8]. Eukaryotic translational elongation factor 2 (eEF2) is an elongation factor which is required for eukaryotic protein synthesis and plays an important role in regulating protein synthesis, catalyzing ribosomes to elongate peptide chains along mRNA [9, 10]. During elongation, eEF2 promotes the translocation of new protein chains from ribosomal acceptor (A) to peptidyl (P) sites in a GTP-dependent manner [11]. By hydrolyzing GTP, eEF2 induces conformational changes in the ribosome complex and promotes translocation, creating an empty A site so that the new tRNA can bind and initiate the next peptide chain extension cycle [12, 13]. Recently, it was reported that increased protein biosynthesis was one of the most important characteristics associated with cancer metabolism [14]. However, few studies have focused on the regulation of protein synthesis in esophageal target therapy. Additionally, although eEF2 was reported as highly expressed in various cancer tissues, the implications of its increased abundance have not been investigated [15]. Thus, clarification regarding the function of eEF2 in ESCC may provide clinically relevant treatment strategies.

Natural compounds are considered as a potential inhibitor repository for screening anti-cancer drugs [16–18]. Toosendanin (TSN), derived from the natural plant Melia toosendan, has ever been used as an anti-parasitic traditional medicine in China [19]. Previous reports showed that TSN could sensitively induce gastric cancer cell apoptosis and suppress the tumor growth of pancreatic cancer [20, 21]. Based on these reports, TSN may exhibit as a potential inhibitor for ESCC. In our previous pull-down results, we identified TSN could bind with eEF2 (Additional file 1). However, the detailed inhibitor effects and underlying mechanisms were not identified.

In the present study, we find that eEF2 plays a positive role during the ESCC cancer process. TSN inhibits ESCC through targeting inhibition of eEF2, thus impedes the downstream proteins synthesis in ESCC. This study supplies a potential therapeutic target and inhibitor for the further ESCC clinical study.

## Methods

### Materials

Toosendanin (Cas:58,812–37-6) was purchased from Weikeqi Biological Technology (Sichuan, China). ESCC tissue array (Cat#HEsoS180Su08) was purchased from OUTDO Biotech (Shanghai, China). The Click-iT® Plus OPP Protein Synthesis Assay Kit was purchased from Invitrogen (Cat#C10456, Carlsbad, CA, USA). The GTPase-Glo Assay kit was bought from Promega (Cat#V7681, Madison, WI, USA). Antibodies to Ki67 (Cat#ab16667), eEF2 (Cat#ab33523), Topoisomerase I (Cat#ab109374), and Topoisomerase II (Cat#ab52934) were purchased from Abcam (Cambridge, MA, USA).

### Cell proliferation assay and colony formation assay

Human ESCC cell lines (KYSE140, KYSE410, KYSE450 and KYSE510) were purchased from the cell bank of National Collection Authenticated Cell Cultures. KYSE140, KYSE410, KYSE450 and KYSE510 cell lines were separated and established from four Japanese who diagnosed with esophageal squamous cell carcinoma. All cell lines were authenticated by STR test prior to expansion and freezing and were free of mycoplasma contamination. Cell lines were cultured in RPMI-1640 media supplemented with 10% fetal bovine serum. The immortalized human embryonic esophageal cell line (SHEE) was a gift from Professor Enmin Li in Shantou University. Cells (1.5–6 × 10^3^ per well) were seeded in 96-well plates and treated with different concentrations of TSN. After incubation for different time points, the cells viability was detected by MTT reagent (0.5 mg/mL). For the colony formation assay, 8 × 10^3^ cells were resuspended in a 0.3% top layer agar over the 0.5% base layer agar. Both the top and base layer agar were mixed with different concentrations of TSN. The imbedded cells were maintained in the cell incubator for 2 weeks. Afterward, the colonies were photographed and counted via Image-Pro Plus v6.0.

### Cell cycle and apoptosis analysis

To evaluate the effect of TSN on cell cycle and apoptosis, 2.5 × 10^5^ cells were seeded in 60 mm dishes containing 0, 5, 10 or 20 nM TSN supplemented media for 48 h or 72 h. In preparation for cell cycle analysis, the cells were fixed in 70% pre-cooled ethanol for 24 h and washed with 1 mL PBS for 3 times. The cells were resuspended with 250 µL 0.5% Triton X-100 and incubated with 5µL 10 µg/mL RNase A at room temperature for 1 h. The cells were then incubated with 5 µL 1 mg/mL propidium iodide at room temperature for 20 min. In preparation for the apoptosis assay, both adherent and suspended cells were harvested and digested gently with trypsin. The cells were then washed with 1 mL chilled PBS and subsequently resuspended with 250 µL Annexin V binding buffer. The cells were then stained with 5 µL 1 mg/mL propidium iodide and 5 µL Annexin V-FITC on ice for 30 min. After staining, the cells were analyzed using a flow cytometer (BD Biosciences, San Jose, CA).

### Vector construction and protein purification

The pcDNA3.1-eEF2-3 × Flag vector (Cat#F122787) was purchased from YouBio company (Hunan, China). The eEF2 fragment sequences (F1- F5) were cloned from pcDNA3.1-eEF2-3 × Flag vector using the EcoR I and BamH I enzymes. Fragments F1, F2, F3 and F4 were individually constructed with pcDNA3.1–3 × Flag vector. Fragment F5 was ligated into the p3 × Flag-cmv14 vector. 120 ng fragment was ligated with 60 ng vector in 5 µL solution I (TAKARA) ligation buffer at 25℃ for 2 h, the mixture was then transferred into DH5ɑ competent cells and identified by sequencing. The constructed vectors were transfected with a concentration of 5 µg per dish into 293 T cells for protein purification by using jetPRIME Transfection Reagent (Polyplus, New York, USA). After transfection for 48 h, 10 dishes 293 T cells were collected and resuspended in 4 mL RIPA lysis buffer over ice. After centrifuging, the cell lysates were co-incubated with 50 µL Flag-beads at 4 ℃ for 15 h. The Flag-beads were washed four times using 1 mL pre-cooled TBS buffer followed by centrifugation at 6000 g for 30 s at 4℃. After washing, the beads were eluted four times using 100 µL 125 μg/mL 3 × Flag peptide buffer in a 4℃ cold room every 40 min. Finally, the proteins were condensed to about 100 µL 200 ng/µL by Amicon Ultra tube (Millipore) and concentrations were measured by BCA kit.

### Lentiviral infection and transfection

5 µg packaging vectors (pMD2.G, psPAX2) were co-transfected with 5 µg pLKO.1-mock or sheEF2, shTOP1 and shTOP2 into 293 T cells using 30 µL jetPRIME Transfection Reagent. After 48 h, the virus particle enriched media was harvested and filtered through 0.45 μm filters. Target cells were then incubated with 1 mL the filtered media supplemented with 8 μg/mL polybrene (Millipore, Billerica, MA) overnight. The next day, the cells were subjected to selection using 4 μg/mL puromycin for 48 h. The remaining viable cells were subsequently utilized for proliferation experiments. For virus treatment of PDX mouse model, harvested virus was subsequently condensed via ultracentrifugation. 40 mL of virus was added into centrifuge tubes and centrifuged at 30,000 rpm for 3 h at 4℃. After centrifugation, the virus was resuspended in 0.4 mL of PBS and each tumor-bearing mouse was infected with 100 μL of the virus.

### Virus concentration detection

The concentration of virus was detected according the Guidelines of Lenti-X p24 Rapid Titer Kit (Cat# 632,200, TAKARA). After the titer kit warmed at room temperature for 30 min, 20 µL of virus lysate was added into the ELISA plates, and then incubated with 200 µL of standards or virus samples at 37℃ for 60 min. After washing by 350 µL 1 × washing solution for 5 times, 100 µL of Anti-p24 (Biotin conjugate) antibody was added into each well and incubated at 37℃ for 60 min. After 5 times washing, the well was incubated with 100 µL Streptavidin-HRP at room temperature for 30 min followed by dispensing 100 µL of Substrate Solution into each well and incubating 30 min without light. 30 min later, 100 µL of Stop Solution was added and the absorbance was measured at 450 nm immediately.

### Molecular docking model

The molecular docking assay was conducted to assess whether TSN could bind with eEF2. The eEF2 crystal structure was downloaded from the protein databank (PBD: 1N0U). The structure was prepared for the docking using the standard settings of the Protein Preparation Wizard (Schrödinger Suite 2015). The hydrogen atoms were set to pH 7.0 after the removal of water molecules in the eEF2 crystal structure. TSN was docked to eEF2 in accordance with the LigPrep default parameters.

### Surface Plasmon Resonance (SPR)

SPR was performed using the Biacore T200 instrument as previously described [22]. eEF2 protein was diluted in 10 mM sodium acetate (pH 4.5) at a concentration of 15 μg/mL for immobilization. After immobilizing about 4 µg eEF2 protein on a CM5 chip (Cat#BR-1005–30, GE Healthcare), TSN or DDD107498 (dissolved in PBS with 0.1% DMSO) were perfused through the ligand channels at different concentrations ranging from 10 nM to 1 µM. The T200 evaluation software was used to analyze the binding affinity between eEF2 with TSN or DDD107498.

### Cellular thermal shift assay (CETSA)

Cellular thermal shift assay was performed as previously described [23]. Forty-eight hours after transfecting the pcDNA3.1-eEF2-3 × Flag vector into 293 T cells, the cells were treated with 200 nM TSN or DMSO for 1 h. The cells were harvested in PBS and divided into several groups (100 µL per group) according the individual temperatures. After heating at different temperatures for 3 min, cells were subjected to two freeze–thaw cycles. After quantification of protein concentration, the supernatant fractions were subjected to Western blotting analysis. The overall thermal shift value was calculated using a sigmoidal curve fit.

### Isothermal Titration Calorimetry (ITC) assay

Micro-Cal PEAQ-ITC (Malvern Panalytical, USA) was used to measure the thermodynamic parameters variation during TSN interaction with eEF2. The sample cell was added with 0.5 µM TSN solution resolving in 300 µL PBS with 0.1% DMSO, while the sample cell reference cell was filled with 300 µL deionized water. The syringe was automatically filled with 70 µL 5 µM eEF2 solution (1 × PBS with 0.1% DMSO) by the machine. During the measurements, a total 19 injections (first injection 0.4 µL, the left injections 2 µL each) were titrated into the sample tank at 150 s intervals. The stirring speed and reference power were set at 750 rpm and 5 μcal·s^−1^, respectively. The results were analyzed by MicroCal PEAQ-ITC Analysis Software.

### Protein synthesis assay

After treatment with 0, 5, 10 or 20 nM TSN for 24 h, the nascent synthesized proteins were labeled with 10 µM Click-iT® OPP for another 30 min according the manufacturer’s specifications of Protein Synthesis Assay Kit [24, 25]. After fixed by 3.7% formaldehyde for 15 min, the cells were subsequently stained by 100 µL OPP reaction cocktail and NuclearMask™ Blue. The stained cells were imaged using an Olympus System Microscope and analyzed by Image-Pro Plus v6.0.

### GTPase assay

Purified eEF2-Flag proteins were used for conducting the GTPase assay. After co-incubation of eEF2 with 0, 10, 20, or 40 nM TSN for 15 min, 12 µL GTPase/GAP buffer was added into the tube and incubated for 1 h at 30℃. After incubation, the reaction complexes were centrifuged at 12,000 g for 5 min and the supernatants were transferred into new tubes. Next, 25 µL reconstituted GTPase-Glo™ reagent component (500 × GTPase-Glo™ Reagent and 10 mM ADP in GTPase-Glo™ Buffer) were added into each tube and incubated with shaking for 30 min at room temperature (22–25°C). After incubation, the liquids were transferred into a white 96-well plate and mixed with 50 µL/well detection reagent. The results were measured by Luminoskan Ascent (Thermo).

### Western blotting

Harvested cells or tissues were lysed using RIPA lysis buffer. After incubating on ice for 30 min, the lysates were centrifuged at 12 000* g* for 10 min and the supernatant was transferred to a fresh tube. The BCA kit (Cat#PC0020, Solarbio) was used to quantify protein concentration in each harvested lysate. After running gel electrophoresis, the proteins were transferred to PVDF membranes. The membranes were subsequently blocked with 5% non-fat milk following by incubation with eEF2, TOP1, TOP2, β-actin or GAPDH antibodies. After incubation with the secondary antibody, the proteins were detected by incubating the membranes in BeyoECL reagent (Cat# P0018S, Beyotime). The membranes were exposed using Amersham Imager 600.

### RNA-binding protein immunoprecipitation sequencing (RIP-Seq)

RNA-binding protein immunoprecipitation was performed using the EZ-Magna RIP Kit (Cat#17–701, Millipore) according the manufacturer’s specifications. After pre-washing 50 µL protein A/G Magnetic Beads with PBST, 400 µL washing buffer with 4.5 µg IgG or eEF2 antibody were added into the tubes and rotated for 2 h in a 4℃ cold room. After incubation, the beads were washed three times and incubated with 3 mg (total volume 1 mL) quantified cell lysates with DMSO or 200 nM TSN at 4℃ for 15 h. The beads were subsequently washed five times and prepared for RNA purification. RNA concentration was measured using a Nanodrop (Thermo). The purified RNA products were subsequently used for RT-PCR or depleted of rRNA using the TIANSeq rRNA Depletion Kit (Cat#NR101, TIANGEN) prior to RNA sequencing.

### Real-time PCR

After extracting RNA by TRIzol regent according to the manufacturer’s instructions, cDNA was synthesized via applying PrimeScript RT reagent Kit (Cat#RR047A, TAKARA). TOP1 and TOP2 relative mRNA levels were then detected through quantitative Real-time PCR (Cat#RR420A, TAKARA). The primers used for the reactions as bellow: TOP1: 5’-GCTTCTCTAGTCCACCACAAA-3’ and 5’-ATCAGC ATCATCCTCATCTCG-3’; TOP2: 5’-GGTGAGATGGAACTCAAGCC-3’ and 5’-GCTCTTCTGACCATTAGTGCA-3’; GAPDH: 5’- CAGCCTCAAGATCATCAGCA-3’ and 5’-TGTGGTCATGAGTCCTTCCA-3’.

### Patient derived xenograft (PDX) mouse model

Six-week old female Non-obese diabetic/severe combined immunodeficient (NOD/SCID) mice were purchased from Vital River Labs (Beijing, China) and raised in a pathogen-free 12 h light/dark cycle environment. All animal procedures were approved by the Ethics Committee of China-US (Henan) Hormel Cancer Institute. The clinical samples were obtained from cancer patients that provided informed consent. ESCC tissues were obtained from Henan Cancer Hospital and passaged for another 3 generations prior to use. The PDX tumor tissues were cut into 0.10–0.12 g fragments and implanted into the right flank of each mouse. After the tumor volume reached approximately 100 mm^3^, the mice were randomly divided into different groups. The detailed information regarding each PDX case is provided in Supplementary Fig. 1. For lentivirus treatment groups, the implanted PDX tissues were directly injected with 100 µL condensed sh-mock, sh-eEF2-2, sh-eEF2-3 virus every three days over a 12-day time period. Vehicle (5% DMSO and 20% PEG400 in PBS), TSN (5 mg/kg, 20 mg/kg) or DDD107498 (20 mg/kg) were administered by oral gavage once daily. The tumor volumes were measured twice per week. When the tumor volume reached approximately 1 cm^3^, the tumor tissues were harvested after sacrificing the mice.

### Immunohistochemistry (IHC) assay

Tumor tissues were embedded in paraffin and cut into slices prior to IHC staining. The tissues were subjected to antigen retrieval and blocking according the Rabbit SP Detection Kit (Cat#SP9001, ZSGB-BIO). The tissue slices were incubated with 50 µL eEF2, Ki67, TOP1 and TOP2 primary antibodies at 4℃ for 15 h. The slices were then washed and incubated with secondary antibodies at room temperature for 30 min. The slices were then stained with 3,3'-Diaminobenzidine (DAB) working buffer. After re-staining with hematoxylin, the slices were photographed and analyzed using the Aperio ImageScope software.

### Statistical analysis

All statistical analyses were conducted using the GraphPad Prism 7.0 software. The paired and unpaired Student’s *t*-test or ANOVA were used to assess significant differences (*p* < 0.05). The results were expressed with mean ± SD.

## Results

### eEF2 protein is highly expressed in ESCC patient tissues and indicated a poor clinical prognosis

**Fig. 1:**
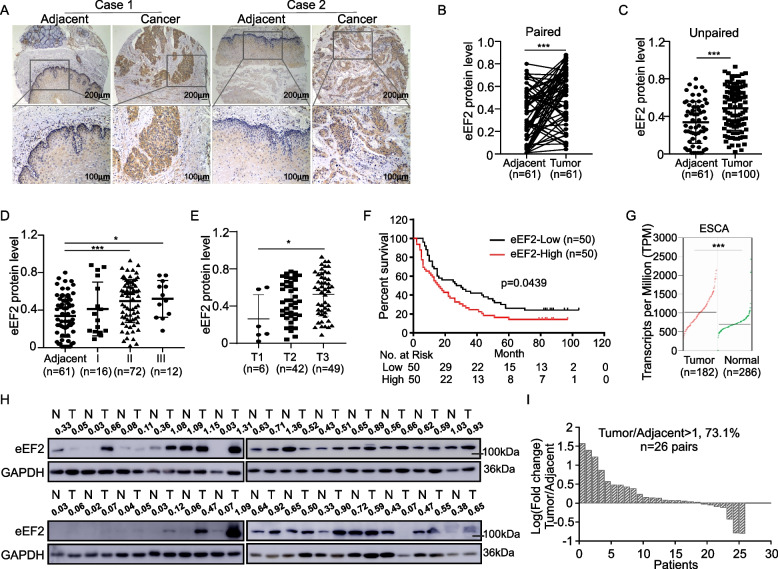
Highly expressed eEF2 predicts poor prognosis in ESCC patients. **A** Representative immunohistochemical staining images of eEF2 in ESCC tissue array. **B** Analysis of eEF2 protein levels in paired tissues. **C** Analysis of eEF2 protein levels in unpaired tissues. **D** Analysis protein level of eEF2 in different pathological grading. **E** Analysis protein levels of eEF2 in different clinical stage. Due to some clinical stage information were missing, the case number was not same with total. **F** Relationship between eEF2 protein level and overall survival from ESCC tissue array. **G** Summarize of eEF2 expression levels in normal or esophageal carcinoma tissues base on GEPIA2 database. **H** eEF2 protein levels in 26 paired ESCC tissues, performed by Western blot. **I** Waterfall plot showing fold-change expression of eEF2 in the 26 ESCC samples as compared with paired adjacent tissues. Statistical analysis was performed using Student’s paired *t* test in (B); Student’s unpaired *t* test in (C, D, E, G); Kaplan–Meier analysis was used in (F). Error bars represent the mean ± SD

We first measured eEF2 protein expression levels using IHC staining across the patient samples included on a tissue array. The representative images were illustrated in Fig. 1A. The IHC staining results indicated that eEF2 protein levels in ESCC tumor tissues were higher than adjacent tissues (Fig. 1B, C). To evaluate the clinical significance of eEF2 in ESCC, we measured the correlation of eEF2 protein expression levels with tumor pathological grading, stage, and prognosis. Based on the clinical information provided with the tissue array (Table 1) and the IHC staining results, we identified an elevated correlation between eEF2 and pathologically graded (II/III) samples compared to that observed in adjacent tissues (Fig. 1D). eEF2 protein levels in clinical stage 3 tissues were also significantly higher than protein levels observed in clinical stage 1 tissues (Fig. 1E). These results indicated that increased eEF2 protein levels are correlated with worse clinicopathologic grade and staging. Furthermore, the results indicated that overall survival rate of ESCC patients with increased eEF2 protein levels is significantly lower (Fig. 1F). In addition, we used the GEPIA2 database to evaluate whether eEF2 was also highly expressed in the TCGA-ESCA patient cohort. The findings indicated that eEF2 mRNA levels were also increased in patient tumors compared to normal tissues (Fig. 1G). Next, we measured eEF2 protein levels in 26 pairs of ESCC tissues and cell lines using Western blot. Our results showed that 18 out of 26 tissue pairs exhibited increased eEF2 protein levels compared to adjacent tissues (Fig. 1H). The gray value intensities indicated that eEF2 expression was significantly increased in 73.1% of primary ESCC tissues compared to the respective adjacent tissues (Fig. 1I). Moreover, compared with the SHEE immortalized human esophageal epithelial cell line, eEF2 was also highly expressed in most ESCC cell lines (Supplementary Fig. 2). In sum, our findings suggest that eEF2 is a potential prognosis index for ESCC patients.

**Table 1:** Cohort characteristics of esophageal cancer patients

Clinicopathological characteristics	eEF2 protein expression level		
	**Low (*n* = 50)**	**High (*n* = 50)**	***P***
Gender			
Male	33 (66%)	40 (80%)	0.010
Female	17 (34%)	10 (20%)	
Age			
≤ 60	16 (32%)	16 (32%)	0.672
> 60	33 (66%)	34 (68%)	
Histological grade			
I	11 (22%)	5 (10%)	0.176
II	33 (66%)	39 (78%)	
III	6 (12%)	6 (12%)	
Clinical stage			
1	3 (6.0%)	3 (6.0%)	0.019
2	26 (52%)	16 (32%)	
3	21 (42%)	28 (56%)	
Tumor size(mm^3^) (Mean ± SD)	22.53 ± 3.621	25.83 ± 3.92	0.538
Lymph node (Mean ± SD) Positive lymph node	8.09 ± 0.81	8.47 ± 1.02	0.771
(Mean ± SD)	1.83 ± 0.45	1.52 ± 0.23	0.548

Numbers do not equal to the total number due to missing data

### eEF2 promotes cell proliferation of ESCC

**Fig. 2:**
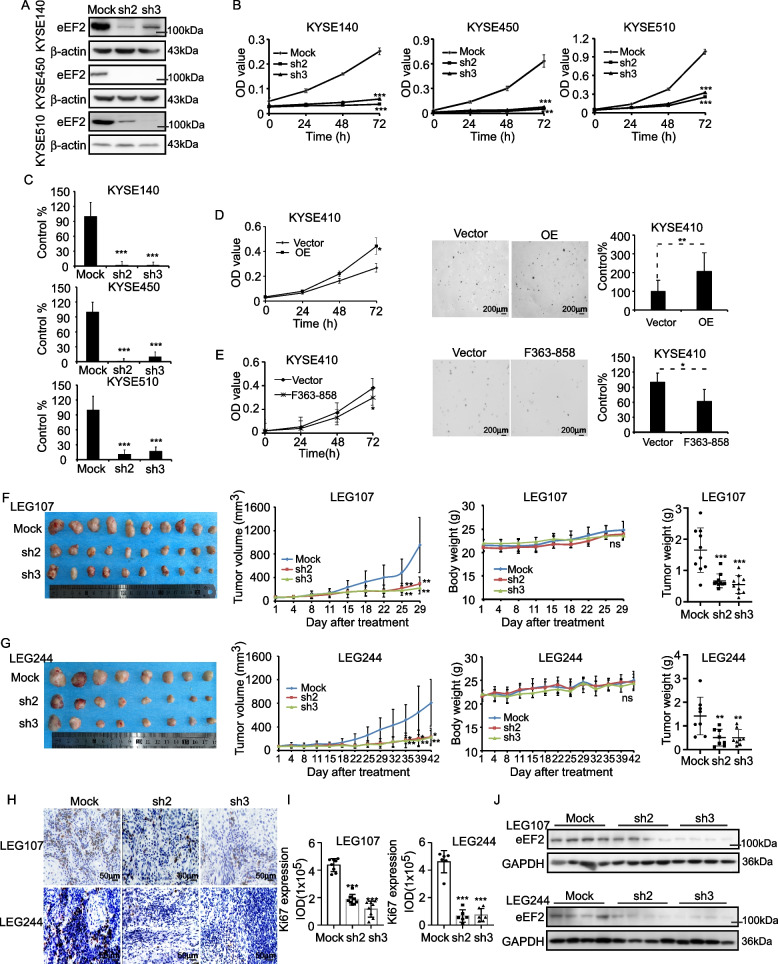
eEF2 was a potent target in ESCC. **A** eEF2 protein levels in knockdown cell lines. **B** Cell viability of KYSE140, KYSE450 and KYSE510 after knockdown of eEF2. **C** Colony formation of ESCC cell lines after knockdown of eEF2. **D** Cell viability and colony formation of KYSE410 after eEF2 overexpressed. **E** Cell viability and colony formation of KYSE410 after eEF2 fragment F363-858 overexpressed. **F**&**G** The tumor volumes, representative pictures, average body weight and tumor weights of LEG107 and LEG244 PDX cases after infected by eEF2 shRNA virus. **H** Immunohistochemical staining of ki67 in tumor slices. **I** Ki67 expression level in (H). **J** eEF2 protein levels after knocking down of eEF2 in PDX models. Student’s unpaired *t* test in (B, C, D, E, F, G, I). Error bars represent the mean ± SD

To evaluate the molecular function of eEF2 in ESCC, we silenced eEF2 in KYSE140, KYSE450 and KYSE510 cell lines which highly expressed eEF2 compared to the other cell lines by shRNA virus and verified silencing efficiency by Western blot. The concentration of shRNA virus was measured by Lenti-X p24 Rapid Titer Kit and was shown in Supplementary Fig. 3A. Results showed that eEF2 protein levels obviously decreased after silencing eEF2 (Fig. 2A). We next conducted an MTT assay to measure the effect of eEF2 knockdown on the cell viability of KYSE140, KYSE450, and KYSE510. Our results indicated that cell viability significantly deceased after eEF2 knockdown (Fig. 2B). Moreover, clonogenic assay results indicated that anchorage-dependent growth was reduced in KYSE140, KYSE450 and KYSE510 following eEF2 knockdown (Fig. 2C; Supplementary Fig. 3B). We also over-expressed eEF2 in the KYSE410 cell line, which exhibited lower eEF2 protein levels compared to the other cell lines, to characterize the effect of increased eEF2 expression on cell viability. Our results indicated that both the cell viability and colony formation ability significantly increased following eEF2 over-expression (Fig. 2D; Supplementary Fig. 4A). In addition, we overexpressed F363-858 fragment of eEF2, which lacking the GTPase domain, to evaluate the effect of eEF2 on cell growth under GTPase activity deficiency in KYSE410. Results showed that overexpressing F363-858 in KYSE410 slightly decreased the cell viability and colony number (Fig. 2E; Supplementary Fig. 4B). These results suggested that eEF2 expression was positively correlated with ESCC cell proliferation. Furthermore, we evaluated the effect of silencing eEF2 in vivo using a lentivirus intra-tumoral infection PDX murine model. After treated with the condensed virus, the average tumor volume and weight in the LEG107 and LEG244 cases were suppressed after silencing eEF2, while the average body weight of mice was not significantly changed (Fig. 2F, G). IHC staining results indicated that Ki67 protein levels were reduced in tumors after eEF2 knockdown, indicating a significant reduction in tumor proliferation (Fig. 2H-J).

### TSN binds with eEF2 and inhibits its GTPase activity

**Fig. 3:**
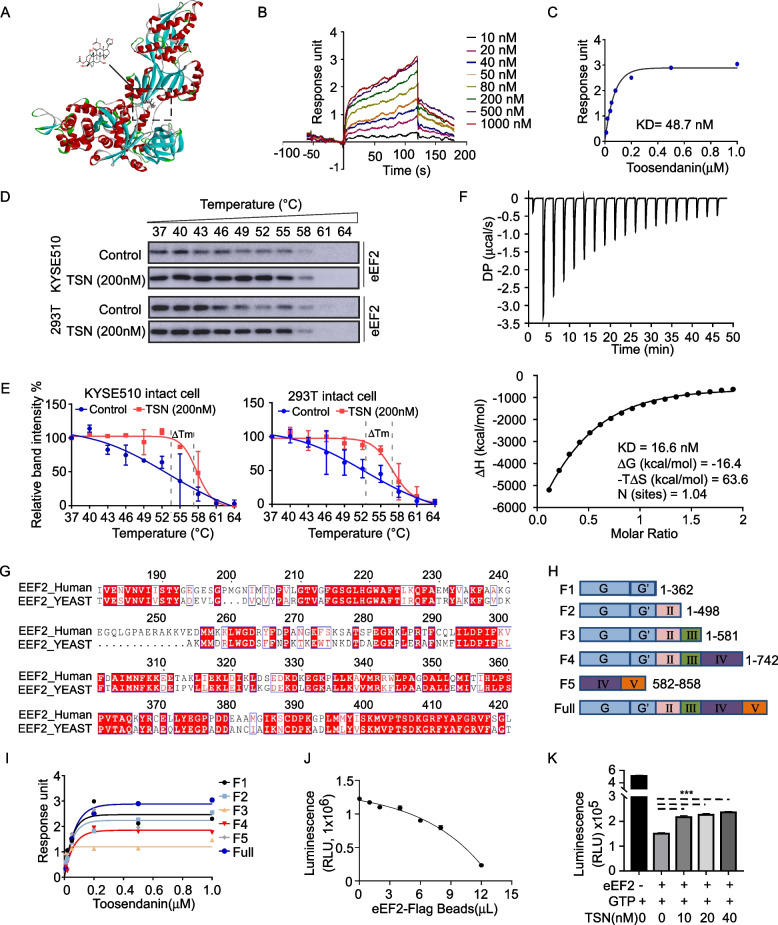
Toosendanin (TSN) is an inhibitor of eEF2. **A** Computational docking model between eEF2 and TSN. **B** The change of response intensity between TSN and eEF2 with the passage of time, checking by SPR. **C** The variation of response intensity with the difference concentration of TSN. **D** The Western blot band of eEF2 for cellular thermal shift assay. **E** The relative band intensity of eEF2 in control and TSN treatment group. **F** ITC profiles and binding curves for the binding of TSN with eEF2. **G** Protein sequence similarity analysis of eEF2 between yeast and human. **H** The diagram of different fragments of eEF2. **I** The binding ability of TSN with different fragments, checking by SPR. **J** The GTPase activity of purified eEF2 protein. **K** TSN inhibits the GTPase activity of eEF2. Student’s unpaired *t* test in (K). Error bars represent the mean ± SD

In the Fig. 2, results have indicated that eEF2 plays a crucial role in ESCC tumor maintenance. We next screened the compound repository in our laboratory to discover an inhibitor of eEF2. The molecular docking assay indicated that TSN could bind with eEF2 (Fig. 3A). We also confirmed binding between eEF2 and TSN by SPR in vitro. The results showed that the response unit increased in a TSN concentration-dependent manner; the dissociation constant (KD) value was calculated as 48.7 nM (Fig. 3B, C). In addition, we also explored whether eEF2 was a target of TSN in KYSE510 and 293 T cells over-expressing eEF2 using a cellular thermal shift assay (CETSA). The CETSA results showed that TSN stabilized the melting temperature (Tm) of eEF2 with the curve shifting about 2.99℃ and 3.11℃ in KYSE510 and 293 T cell lines, respectively. These findings suggested that TSN directly binds to eEF2 in intact cells (Fig. 3D, E). Furthermore, ITC assay was conducted based on the variation of thermodynamic during the interaction. The ΔH and -TΔS results implied that TSN bind with eEF2 via hydrogen, ionic and van der Waals force interacts rather than hydrophobic interaction. The calculated data showed the KD value of TSN was about 16.6 nM which confirmed TSN could bind with eEF2 (Fig. 3F). To identify the eEF2 domain responsible for facilitating its interaction with TSN, we truncated eEF2 to five fragments according to the structure of eEF2 in yeast (Fig. 3G, H). Different fragments of eEF2 were cloned into pcDNA3.1–3 × Flag or p3 × Flag-cmv14 vectors; the enzyme restriction identification analysis is provided in Supplementary Fig. 5A. We individually transfected the plasmid constructs into 293 T cells and verified the protein expression of truncated eEF2 (Supplementary Fig. 5B). Purified proteins isolated from the 293 T cells expressing the eEF2 fragments were directly utilized for SPR analysis (Supplementary Fig. 5C). The results of the SPR analysis indicated that TSN bound with all fragments aside from F5, which lacks the GG’ domain (Fig. 3I). As eEF2 is a GTPase, we next detected whether TSN affects its activity using a GTPase kit. The results showed that the GTPase activity of eEF2 increased in a dose-dependent manner (Fig. 3J). After adding different concentrations of TSN to eEF2, the results showed that TSN could indeed inhibit its GTPase activity (Fig. 3K).

### TSN inhibits cell proliferation and colony formation ability of ESCC cell lines

**Fig. 4:**
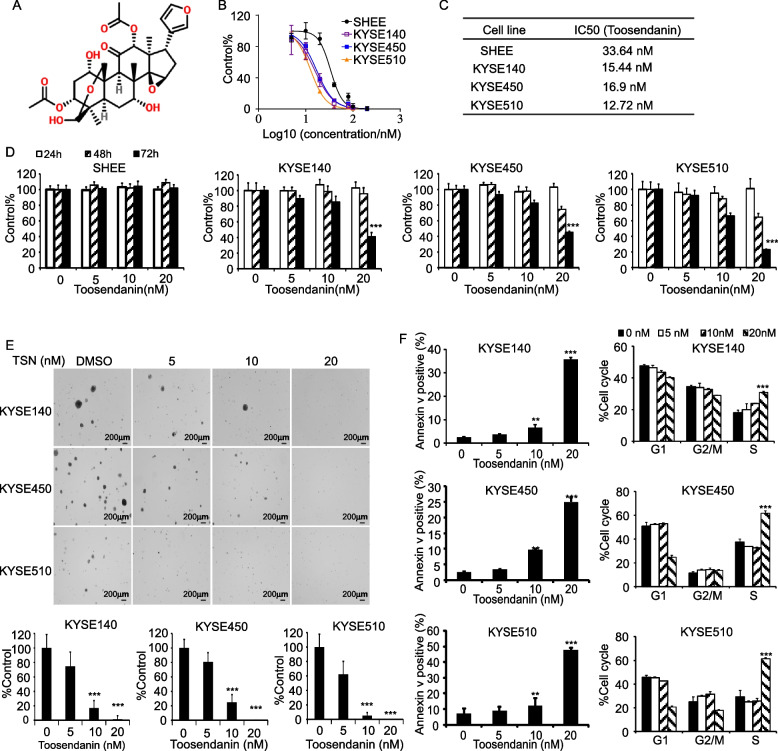
TSN inhibits ESCC cell proliferation. **A** Chemical structure of TSN. **B**&**C** The analysis IC_50_ of TSN on SHEE, KYSE140, KYSE450, KYSE510 by cell viability assay. **D** The cell viability of SHEE, KYSE140, KYSE450, KYSE510 after treated by 0, 5, 10, 20 nM TSN. **E** Colony formation of KYSE140, KYSE450 and KYSE510 after TSN treatment. **F** The cell cycle and cell apoptosis states after TSN treatment. Student’s unpaired *t* test in (D, E, F). Error bars represent the mean ± SD

In the Fig. 3, results showed that TSN was a potent inhibitor of eEF2 in vitro. We next selected a wide range concentration (0–200 nM) of TSN to evaluate its inhibitory effects on ESCC cell lines. The chemical structure of TSN is shown in Fig. 4A. Next, cell viability and IC_50_ were measured by MTT assay after treating cell lines for 72 h with TSN (Fig. 4B). The results indicated that the IC_50_ of TSN in the SHEE cell line was much higher than ESCC cancer cell lines, which indicated that TSN had less toxicity on normal esophageal cell (Fig. 4C). Therefore, we selected the 0, 5, 10, 20 nM TSN concentrations to conduct the subsequent experiments. Results showed that TSN could inhibit the proliferation of the KYSE140, KYSE450 and KYSE510 cell lines at 20 nM; however, no significant effects on the proliferation of the SHEE cell line were observed at the same concentration (Fig. 4D). Moreover, the colony formation ability of the KYSE140, KYSE450 and KYSE510 cell lines significantly decreased after treatment with TSN (Fig. 4E). We next sought to determine the effect of TSN treatment on cell apoptosis and cell cycle progression. The results showed that TSN could induce apoptosis and promote S phase cell cycle arrest in KYSE140, KYSE450, and KYSE510 (Fig. 4F). Moreover, we also rescued eEF2 expression in eEF2 silenced cells and treated with TSN to assess whether its inhibitory effect was rescued or not. The cell viability results showed that the inhibitory effects of TSN were partly rescued after eEF2 overexpressed in eEF2 silenced cells (Supplementary Fig. 6A). Similarly, the colony formation inhibitory ability of TSN was also enhanced after eEF2 rescued (Supplementary Fig. 6B). Overall, these data indicated TSN inhibited ESCC cell lines proliferation through targeting eEF2.

### TSN inhibits TOP1 and TOP2 protein synthesis in ESCC cells

**Fig. 5:**
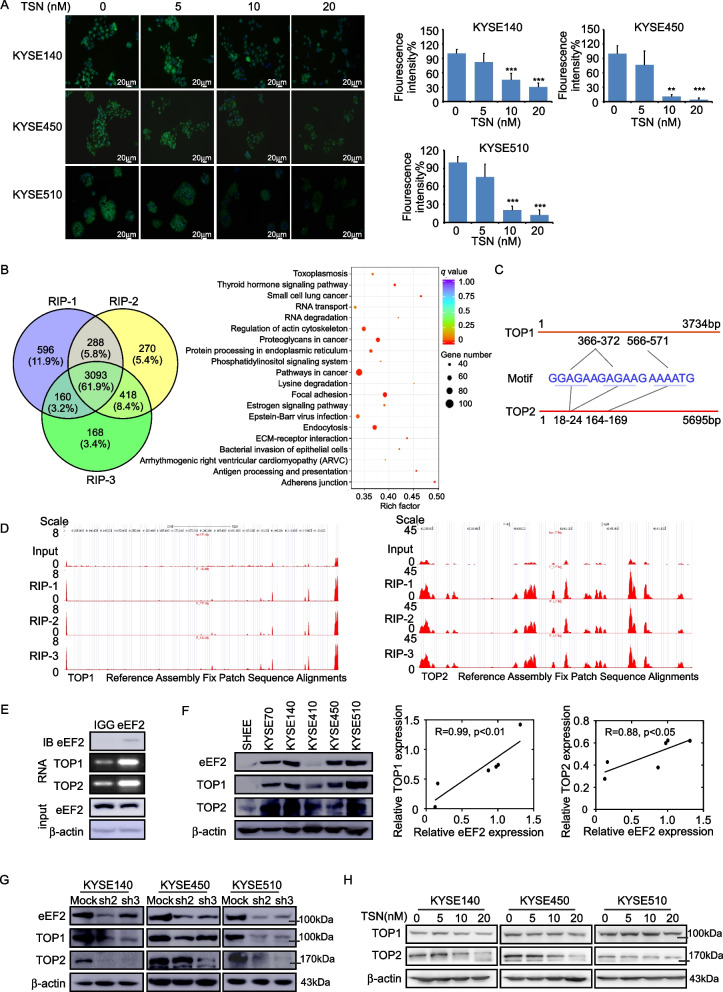
eEF2 regulates the protein biosynthesis of TOP1 and TOP2. **A** Represent pictures and analysis of protein biosynthesis levels after TSN treatment. **B** left: the merged gene of three replicates RIP-sequence results; right: the enriched KEGG pathways of the merged genes. **C** The motifs of eEF2 targeted on TOP1 and TOP2. **D** The enriched peak levels of TOP1 and TOP2 in the RIP-Sequence. **E** IB, immunoprecipitation for eEF2; RNA, TOP1 and TOP2 mRNA levels after co-immunoprecipitation with eEF2; input, Western blot for the protein levels of eEF2. **F** The protein expression levels and correlation between eEF2 with TOP1 and TOP2 in ESCC cell lines and SHEE cell. **G** TOP1 and TOP2 protein levels after knockdown of eEF2. **H** Protein levels of TOP1 and TOP2 after treating by TSN. Statistical analysis was performed using Student’s unpaired *t* test in (A). Spearman’s nonparametric correlation test performed in (F). Error bars represent the mean ± SD

The previous data suggests that TSN could suppress the proliferation of ESCC cells. Therefore, we sought to determine which biological pathways were altered at the existence of TSN in ESCC cells. A co-immunoprecipitation assay was conducted to identify differential protein banding in untreated and TSN-treated cell lysates. MS analysis of isolated protein bands identified 41 identical proteins and 85 different proteins between the two groups. We next used the DAVID Bioinformatics Suite to explore which biological processes were potentially affected by TSN treatment. The top eight differential biological processes are summarized in Supplementary Fig. 7A. The results of the analysis indicated that the protein translation process was the highest affected biological process in the both proteins groups. This result indicated the inhibition of eEF2 by TSN potentially modulates protein synthesis in ESCC. We next conducted a protein synthesis assay on untreated and TSN-treated ESCC cell lines. We found that the abundance of nascently synthesized proteins in KYSE140, KYSE450 and KYSE510 were reduced after TSN treatment (Fig. 5A). Additionally, we utilized RIP-Sequence to identify which translation products were regulated by eEF2. The results indicated that a total of 3093 transcripts were associated with eEF2 protein. These transcripts were subsequently queried using KEGG enrichment analysis. The results of the analysis indicated that the Pathways in Cancer ranked highest of all identified pathways (Fig. 5B). These findings reinforce the important contribution of eEF2 toward regulating the protein synthesis process in cancer. Through the transcripts list we found that TOP1 and TOP2 which were visualized as potential esophageal cancer therapeutic targets were highly enriched compared with the input group [26]. The eEF2 motifs that recognize TOP1 and TOP2 transcript sequences were illustrated in Fig. 5C. The sequenced genome data from the RIP-Seq confirmed that the mRNA of TOP1 and TOP2 were highly enriched by eEF2 (Fig. 5D). Next, we performed RNA-IP assay and quantified mRNA level of TOP1 and TOP2 by real-time PCR to identify whether eEF2 binds with them or not. The results confirmed that eEF2 could bind with TOP1 and TOP2 transcripts (Fig. 5E). We also found that TOP1 and TOP2 expression were both positively correlated with eEF2 expression in the TCGA-ESCA cohort using the GEPIA2 database (Supplementary Fig. 7B). The TOP1 and TOP2 protein levels were also positively correlated with eEF2 protein levels in ESCC cell lines (Fig. 5F). Furthermore, we found that TOP1 and TOP2 protein levels decreased while the mRNA levels were not significantly changed after silencing eEF2 (Fig. 5G; Supplementary Fig. 7C, D). In addition, we evaluated whether TSN could also affect the synthesis of TOP1 and TOP2. The results showed that TOP1 and TOP2 protein levels decreased significantly after TSN treatment (Fig. 5H); however, their respective mRNA levels were not affected (Supplementary Fig. 7E). Furthermore, we conducted RIP-sequencing with or without the existence of TSN. Results showed that TSN didn’t affect eEF2 binding with the mRNA of TOP1 and TOP2 (Supplementary Fig. 7F; Additional file 2). In sum, these findings implied that TSN affected the protein synthesis of TOP1 and TOP2 through targeting eEF2.

### TOP1 and TOP2 play a positive role in the cell proliferation process

**Fig. 6:**
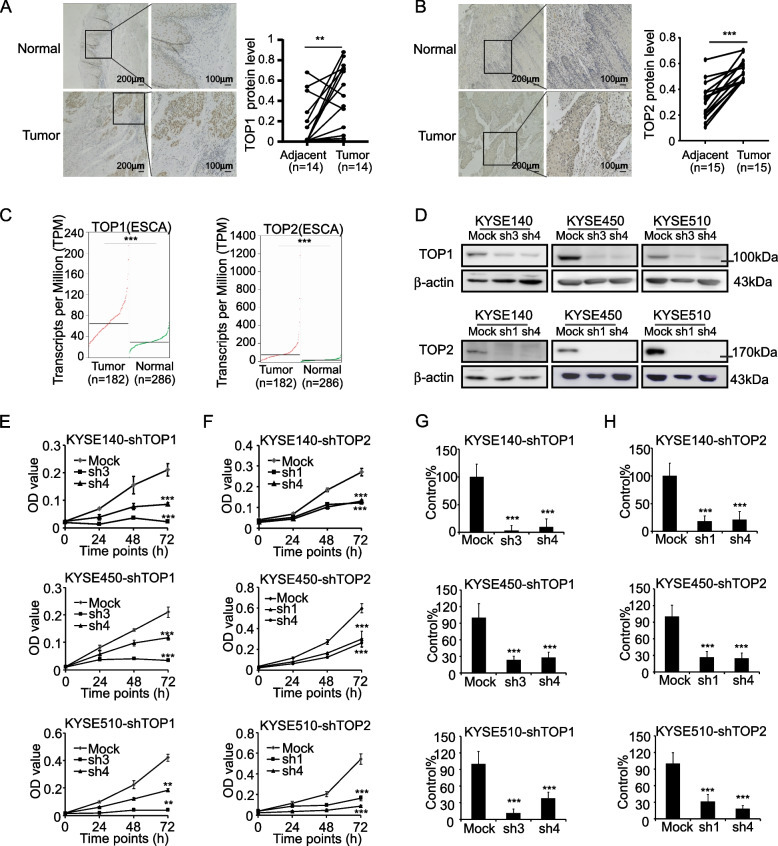
TOP1 and TOP2 promotes cell proliferation in ESCC biological process. **A** Representative IHC staining picture and analysis of TOP1 in ESCC tissues. **B** Representative IHC staining pictures and analysis of TOP2 in ESCC tissues. **C** Analysis of TOP1 and TOP2 expression levels in normal or esophageal carcinoma tissues base on GEPIA2 database. **D** Protein levels of TOP1 and TOP2 in the knockdown cell lines. **E** Cell viability of KYSE140, KYSE450 and KYSE510 after knock down of TOP1. **F** Cell viability of KYSE140, KYSE450 and KYSE510 after knock down of TOP2. **G** Colony formation analysis of KYSE140, KYSE450 and KYSE510 after knock down of TOP1. **H** Colony formation analysis of KYSE140, KYSE450 and KYSE510 after knock down of TOP2. Student’s paired *t* test in (A, B). Student’s unpaired *t* test in (C, E, F, G, H). Error bars represent the mean ± SD

To evaluate the function of TOP1 and TOP2 in ESCC, we checked their protein and transcript levels using ESCC patient tissues and the GEPIA2 database, respectively. IHC staining results showed that TOP1 and TOP2 were highly expressed in ESCC tissues (Fig. 6A, B). The GEPIA2 database also indicated that TOP1 and TOP2 transcript levels were more highly expressed in the tumor tissues than adjacent tissues of the TCGA-Esophageal Cancer cohort patients (Fig. 6C). Similarly, TOP1 and TOP2 were also highly expressed in the gene microarray datasets (GSE23400 and GSE44021) of ESCC (Supplementary Fig. 8A). Moreover, we silenced TOP1 and TOP2 in the KYSE140, KYSE450 and KYSE510 cell lines to evaluate their function in ESCC (Fig. 6D). Results showed that both cell viability and colony number were significantly decreased after silencing TOP1 and TOP2 (Fig. 6E, F, G&H, Supplementary Fig. 8B). In sum, TOP1 and TOP2 were highly expressed and contribute to cell proliferation in ESCC.

### eEF2 regulates ESCC cell growth partly dependent on the expression of TOP1 and TOP2

**Fig. 7:**
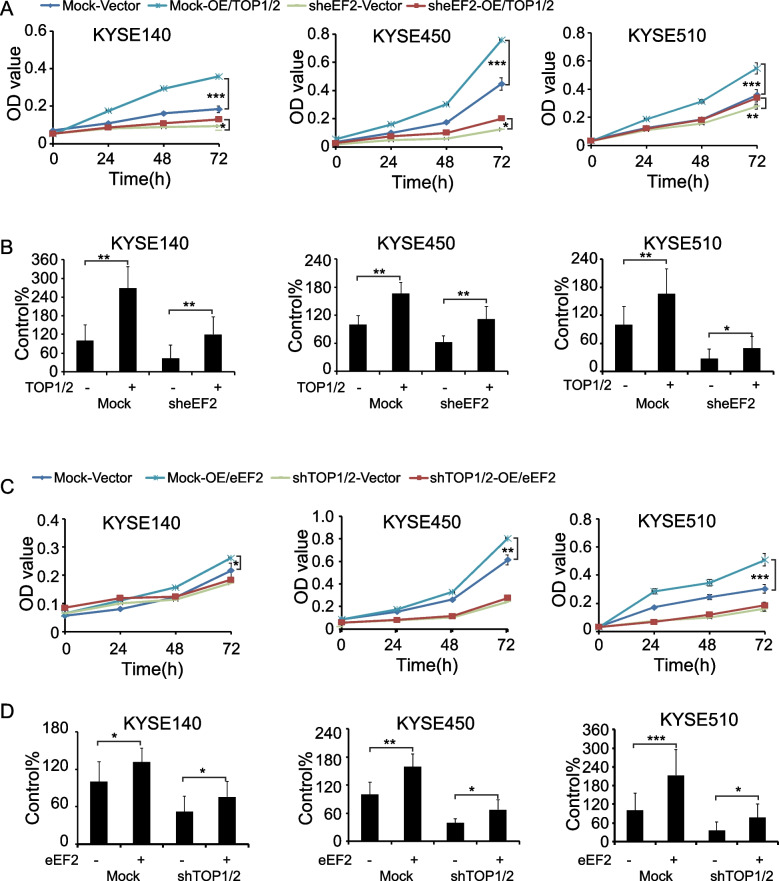
eEF2 promotes cell growth partly through TOP1 and TOP2 in ESCC. **A** The cell viability of KYSE140, KYSE450 and KYSE510 after overexpressed TOP1 and TOP2 in eEF2 silencing cells. **B** The colony formation of KYSE140, KYSE450 and KYSE510 after overexpressed TOP1 and TOP2 in eEF2 silencing cells.** C** The cell viability of KYSE140, KYSE450 and KYSE510 after overexpressed eEF2 in TOP1 and TOP2 silencing cells. **D** The colony formation of KYSE140, KYSE450 and KYSE510 after overexpressed eEF2 in TOP1 and TOP2 silencing cells. Student’s unpaired *t* test in (A-D). Error bars represent the mean ± SD

In order to evaluate whether eEF2 exerted its oncogenic functions through TOP1 and TOP2 in ESCC, we overexpressed TOP1 and TOP2 in eEF2 silenced cells and overexpressed eEF2 in TOP1 and TOP2 silenced cells. After overexpressing TOP1 and TOP2 in eEF2 silenced KEYSE140, KYSE450 and KYSE510 cells, compared with vehicle group, the cell viability and colony formation ability both obviously increased in eEF2 silenced and mock groups (Fig. 7A, B; Supplementary Fig. 9A, B). After overexpressing eEF2 in TOP1 and TOP2 silenced cells, compared with vehicle group, the cell viability slightly increased in KYSE140, KYSE450 and KYSE510 in TOP1 and TOP2 silenced group (Fig. 7C). After overexpressing eEF2 in TOP1 and TOP2 silenced cells, compared with vehicle group, the colony formation ability increased significantly in TOP1 and TOP2 silenced and mock groups (Fig. 7D; Supplementary Fig. 9C, D). Thus, the results indicated that eEF2 exerted its oncogenic functions partly through TOP1 and TOP2 in ESCC.

### TSN suppresses ESCC tumor growth in vivo

**Fig. 8:**
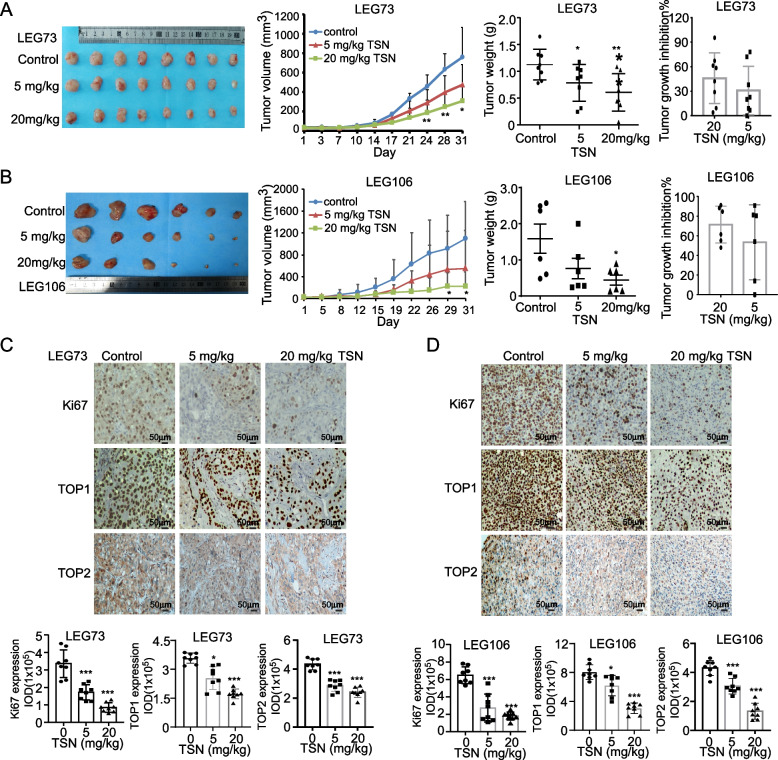
TSN suppresses ESCC PDX mice tumor growth in *vivo*. **A** The analysis of tumor volumes, tumor weights, tumor growth inhibition rate and representative tumor picture of LEG73 after TSN treatment. **B** The analysis of tumor volumes, tumor weights, tumor growth inhibition rate and representative tumor picture of LEG106 after TSN treatment. **C** Represent pictures and analysis of Ki67, TOP1, TOP2 in LEG73 case. **D** Represent IHC staining pictures and analysis of Ki67, TOP1, TOP2 in LEG106 case. Statistical analysis was performed using Student’s unpaired *t* test in (A-D). Error bars represent the mean ± SD

To evaluate the inhibitory effects of TSN on ESCC in vivo, we prepared PDX murine models implanted with tumor tissues (LEG73 and LEG106 cases) to assess changes in tumor volume and weight upon TSN treatment. After sacrificing the mice, we observed that tumor volume and weight in the LEG73 case were significantly decreased after treatment with TSN compared to the vehicle-treated group. The tumor growth of the 20 mg/kg TSN-treated LEG73 group decreased on average by 45.8% (Fig. 8A). Similarly, tumor volume and tumor weight were also significantly decreased in 20 mg/kg TSN-treated mice implanted with the LEG106 case tissues compared to the vehicle-treated group. The tumor growth of the 20 mg/kg TSN-treated LEG106 group decreased on average by 71.4% (Fig. 8B). In addition, the average body weight of mice was not significantly changed compared with the vehicle-treated group, which indicated that TSN had no obvious toxicity at the concentration of 20 mg/kg (Supplementary Fig. 10). Next, we conducted IHC analysis on the excised tumor tissues to measure the protein levels of Ki67, TOP1, and TOP2. The results indicated that the proliferation ability and protein levels of TOP1 and TOP2 were suppressed after treatment with TSN (Fig. 8C, D).

**Fig. 9:**
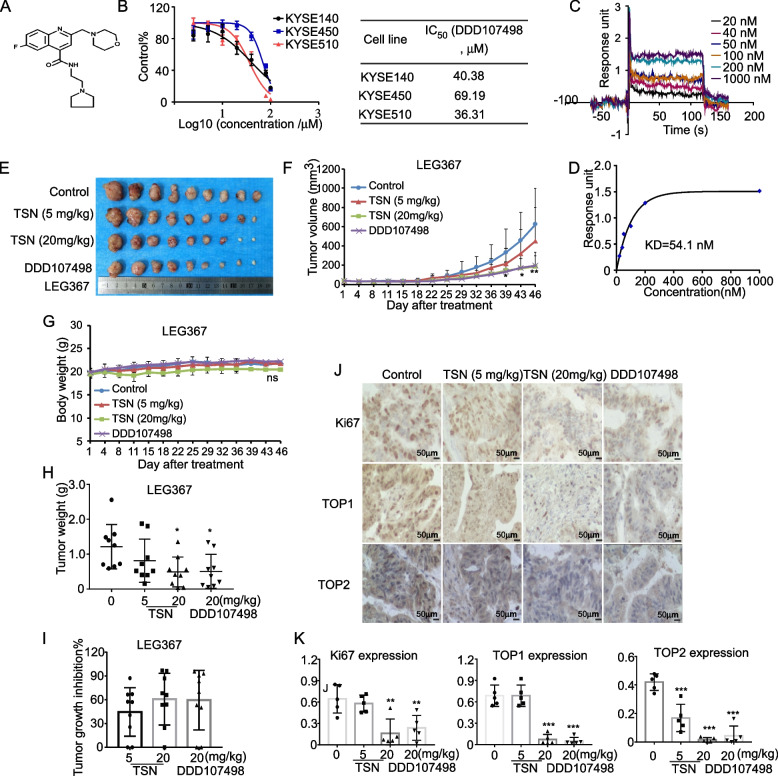
DDD107498 suppresses ESCC growth in *vivo* and in *vitro*. **A** The chemical structure of DDD107498. **B** IC_50_ of DDD107498 base on ESCC cell viability effects. **C** The binding ability between DDD107498 and eEF2 with the passage of time. **D** The variation of response intensity with the difference concentrations of DDD107498. **E** The representative tumor pictures of LEG367 after sacrificing the mice. **F** The tumor volume of LEG367 after treatment. **G** The average body weight of mice after treatment. **H** The tumor weight of LEG367 after treatment. **I** The analysis of tumor growth inhibition rate compared with control group. **J** Represent IHC staining pictures of Ki67, TOP1, TOP2 expression in LEG367 case. **K** Analysis of Ki67, TOP1, TOP2 expression in LEG367 case. Statistical analysis was performed using Student’s unpaired *t* test in (F, H, K). Error bars represent the mean ± SD

DDD107498 was recently reported to be an eEF2 inhibitor [27]. Thus, we sought to compare the inhibitory potential of TSN and DDD107498 in vitro and in vivo. The chemical structure of DDD107498 is illustrated in Fig. 9A. Our results indicated that DDD107498 could significantly inhibit the cell viability of the KYSE140, KYSE450 and KYSE510 cell lines; the IC_50_ of DDD107498 on the three ESCC cell lines was calculated as 40.38, 60.19 and 36.31 µM, respectively (Fig. 9B). We further conducted an SPR assay to verify that DDD107498 could bind with eEF2. Results indicated that DD107498 was able to bind with eEF2; a KD value was calculated as 54.1 nM (Fig. 9C, D). Furthermore, we used DDD107498 as a positive control to treat ESCC PDX case LEG367 to compare its efficacy with TSN in vivo. After sacrificing the mice, the tumors of each group were excised and photographed (Fig. 9E). Both tumor volume and weight were suppressed after treatment of 20 mg/kg of TSN or DDD107498, while the average body weight of mice were not significantly changed in treatment groups (Fig. 9F-H). The tumor growth of 20 mg/kg TSN or DDD107498 treatment group decreased on average by 60.8% and 59.6%, respectively, compared to the vehicle-treated control groups (Fig. 9I). Additionally, IHC staining results showed that Ki67, TOP1 and TOP2 were decreased after mice treated with TSN or DDD107498 (Fig. 9J, K).

## Discussion

Although many compounds have been the subject of preclinical studies in recent years, very few targeted drugs have produced favorable clinical outcomes when applied to ESCC. Protein biosynthesis plays a critical role in cell biological processes. Nevertheless, there few molecular targets which were related to protein translation were developed for the cancer targets therapy. In the present study, we identified eEF2, an important regulator of the protein elongation process, as a potential target of ESCC [28]. eEF2 was reported to be highly expressed in esophageal cancer and endometrial carcinoma; however, its contributing role in these cancers is still unknown [15, 29]. In non-small cell lung cancer, eEF2 was reported to contribute to cancer cell invasion through interacting with arginine methyltransferase 7 [30], yet its detailed molecular function was not deeply investigated. Our results clarified the molecular function of eEF2 and its relationship with ESCC patient prognosis. In addition, we also explored the downstream proteins regulated by eEF2 in ESCC.

In the present study, we found that increased eEF2 protein levels promoted cell proliferation in ESCC. Silencing eEF2 effectively decreased cell proliferation and colony formation ability in ESCC cell lines, suggesting that molecular targeting of eEF2 could be clinically beneficial. However, there are few reports that have actively investigated eEF2 inhibitors in the context of cancer; therefore, we attempted to discover a natural compound inhibitor of eEF2. We identified that TSN was a potential inhibitor of eEF2, although it was reported that TSN could bind with STAT3 in osteosarcoma tumor [31]. Here, TSN have showed more sensitive binding ability with eEF2 than STAT3 according to the KD value. This indicates that TSN has a greater binding affinity towards eEF2.

It was also reported that DDD107498 and sordarin could potentially inhibit eEF2. DDD107498 was found to inhibit eEF2 mediated ribosome translocation along mRNA in mammalian protein synthesis [27]. Sordarin was found to bind to pocket domains III, IV and V of eEF2 in Saccharomyces cerevisiae; however, the GTP hydrolysis activity of eEF2 was not hindered upon binding [32]. Considering that the homologies of yeast and human eEF2 are approximately 67.2% similar, we chose to focus our investigation on DDD107498. The cell viability results showed that DDD107498 inhibited cell proliferation less efficiently than TSN in ESCC cell lines. However, the PDX results highlighted drug response indicated that DDD107498 exhibited similar effects to TSN in vivo. The KD value obtained from SPR assay, was used to measure the binding affinity between eEF2 and TSN or DDD107498 in vitro, while the IC_50_ was used to measure the proliferation inhibitory effect of TSN or DDD107498 in the cell. Thus, the inconsistency between KD value and IC_50_ may be caused by the cell membrane. In this study, DDD107498 was applied to compare the ESCC inhibitory effect with TSN with the same dose 20 mg/kg and this concentration of DDD107498 was also reported to be safety when used for mice [27]. Despite this finding, the results of SPR analysis showed that TSN was a more sensitive inhibitor of eEF2.

eEF2 is a member of the GTPase superfamily and assists the tRNA translocation during protein synthesis [11]. This process may proceed extremely slow without catalyzed eEF2 GTPase activity, which has been found to accelerate translocation by 50-fold [33, 34]. During this process, eEF2 interacts with ribosomal complexes containing tRNA to synthesize nascent peptide chains along the mRNA [35, 36]. Therefore, we performed Co-IP with MS and RIP-Seq analyses to identify which biological processes and mRNA were affected by TSN treatment. According the RIP-Seq results, we found that TOP1 and TOP2 were regulated by eEF2. TOP1 and TOP2 have been widely studied and are considered to be highly relevant targets for the cancer therapy [37–39]. TOP1 was reported to be highly expressed in liver cancer and breast cancer; however, its detailed mechanism has not been fully characterized [40, 41]. Similarly, TOP2 was also recently found to be highly expressed in esophageal cancer through high-resolution and large-scale quantitative proteomic analysis and may be a potential target in esophageal cancer [42, 43]. Thus, it is valuable to explore the molecular function of TOP1 and TOP2 in ESCC. Our results confirmed that TOP1 and TOP2 play a positive role in ESCC tumor maintenance.

**Fig. 10:**
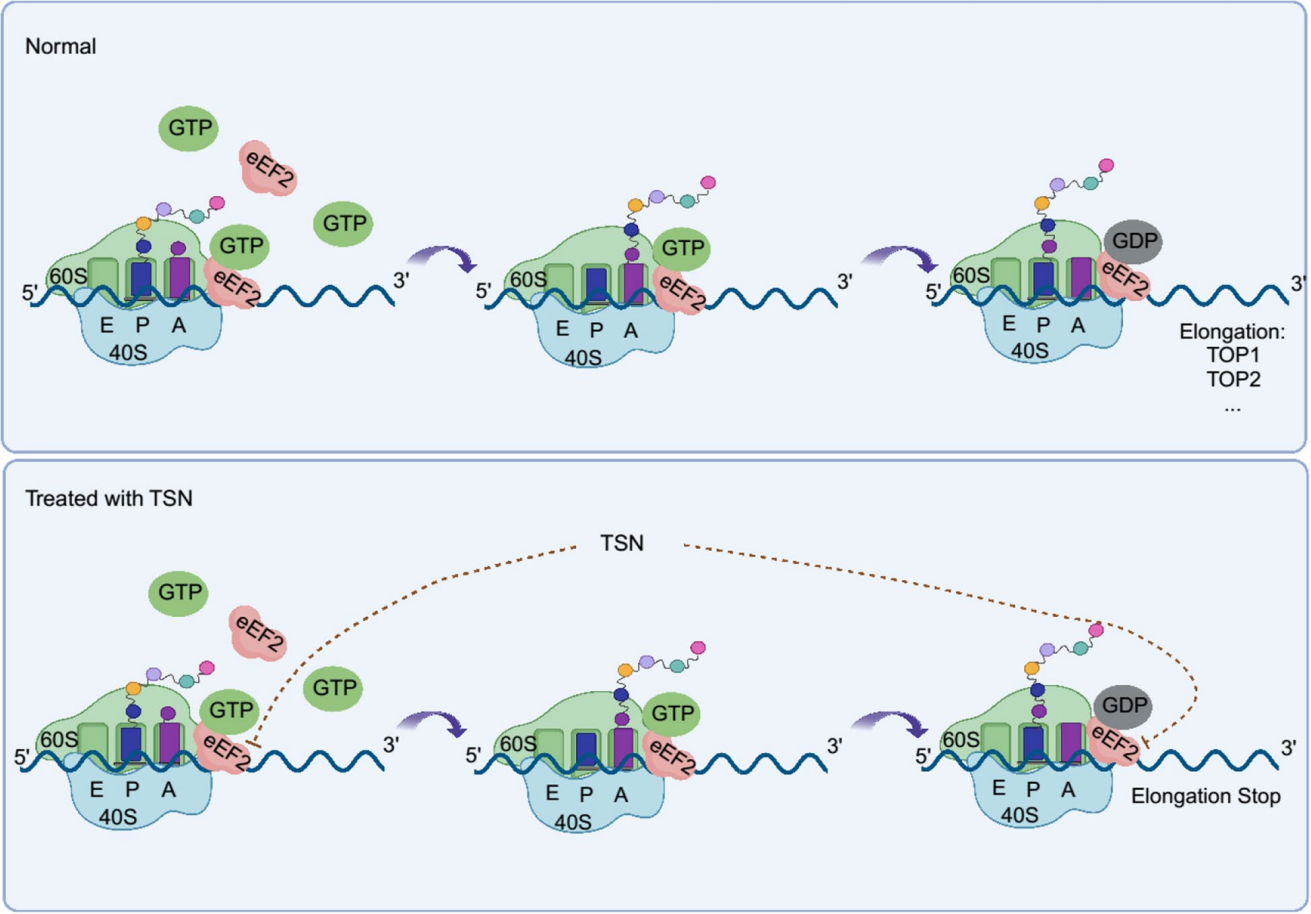
The mechanism schematic diagram of TSN suppressed ESCC growth. First: eEF2 bond with GTP, ribosome and mRNA formed the translation complex; second: the amino which locating at P and A site were hybrid; third: eEF2 hydrolyzed GTP to GDP, resulting its conformation changes, thus ensuring the elongation with the unidirectional mRNA reading frame

Recently, the structure of ribosome-associated eEF2 was precisely visualized at high resolution using cryo-electron microscopy (cryo-EM), solidifying its importance in the intricate translocation process [35, 44, 45]. The translocation process of eEF2 was divided into 5 dynamic steps which accurately clarified its GTP binding and hydrolysis functions during ribosome translocation. Based on the reports, we concluded that TSN competitively binds with eEF2 and inhibits its GTPase hydrolysis activity during the later steps of translocation (Fig. 10). In the present manuscript, there are some potential issues that require further investigation. For example, the PDX mouse model does not accurately reflect the involvement of the immune system in cancer; thus, the influence of TSN on the immune system is presently unknown.

## Conclusions

In summary, we got the conclusions that eEF2 functions as an oncogene and regulates the translation of TOP1 and TOP2 in ESCC. Targeting of eEF2 with TSN inhibits ESCC tumor growth in vitro and in vivo*.* The underlying inhibitory mechanism relies on the inhibition of eEF2 GTPase activity upon binding with TSN. The reduction in GTPase activity subsequently impedes the synthesis of TOP1 and TOP2 proteins, thereby suppressing ESCC proliferation. Targeting on eEF2 impeded protein translation process provided a feasible strategy for the target therapy in ESCC and the proper use of TSN may be benefit for the ESCC patients in the further clinical studies.

## Supplementary Information

**Additional file 1. **MS identified proteins which Toosendanin potentially bond with.

**Additional file 2. **Enriched mRNA list by RIP-sequencing when conducted with DMSO or TSN.

**Additional file 3: Supplement figure 1. **The clinical information of ESCC PDX cases. **Supplement figure 2.** eEF2 protein levels in different ESCC cell lines. **Supplement figure 3.** A The concentration of shRNA virus used in this study. B The representative colony pictures of KYSE140, KYSE450 and KYSE510 after knock down of eEF2. **Supplement figure 4.** Protein expression level in KYSE410. A Western blotting explored the protein expression level in KYSE410 after transfected with eEF2. B. The protein expression level in KYSE410 after transfected with F363-858 fragment. **Supplement figure 5.** The processes of eEF2 construction and purification. A The identification of different clone of fragments by restriction endonuclease. B The protein identification of different fragments by Western blot. C The purification of different fragments checked by comas blue staining. **Supplement figure 6.** Overexpressing eEF2 in eEF2 knockdown cells rescued the inhibitory effects of TSN on ESCC. **Supplement figure 7.** TSN didn’t affect the transcription of TOP1 and TOP2. **Supplement figure 8.** TOP1 and TOP2 expression level and colony pictures. **Supplement figure 9.** Representative colony pictures in KYSE140, KYSE450 and KYSE510 rescued cells. **Supplement figure 10.** The average body weight of mice after treated with TSN in LEG73 and LEG106.

## Data Availability

The RIP-Seq data generated during the current study are available in the [NCBI Sequence Read Archive] repository, [https://submit.ncbi.nlm.nih.gov/subs/bio/project/SUB11991721/overview]. Other data were available in the manuscript [and its supplementary information files].

## Abbreviations

eEF2: Elongation factor 2
ESCC: Esophageal squamous cell carcinoma
ITC: Isothermal Titration Calorimetry
PDX: Patient derived xenograft
SPR: Surface Plasmon Resonance
TOP1: Topoisomerase I
TOP2: Topoisomerase II
TSN: Toosendanin
