# Supplementary material for: Toosendanin targeting eEF2 impedes Topoisomerase I & II protein translation to suppress esophageal squamous cell carcinoma growth
Source: J Exp Clin Cancer Res. 2023 Apr 24;42:97. doi: 10.1186/s13046-023-02666-5 (PMC10124032; doi:10.1186/s13046-023-02666-5)
Supplement: Supplementary file 3 — Additional file 3: Supplement figure 1. The clinical information of ESCC PDX cases. Supplement figure 2. eEF2 protein levels in different ESCC cell lines. Supplement figure 3. A The concentration of shRNA virus used in this study. B The representative colony pictures of KYSE140, KYSE450 and KYSE510 after knock down of eEF2. Supplement figure 4. Protein expression level in KYSE410. A Western blotting explored the protein expression level in KYSE410 after transfected with eEF2. B. The protein expression level in KYSE410 after transfected with F363-858 fragment. Supplement figure 5. The processes of eEF2 construction and purification. A The identification of different clone of fragments by restriction endonuclease. B The protein identification of different fragments by Western blot. C The purification of different fragments checked by comas blue staining. Supplement figure 6. Overexpressing eEF2 in eEF2 knockdown cells rescued the inhibitory effects of TSN on ESCC. Supplement figure 7. TSN didn’t affect the transcription of TOP1 and TOP2. Supplement figure 8. TOP1 and TOP2 expression level and colony pictures. Supplement figure 9. Representative colony pictures in KYSE140, KYSE450 and KYSE510 rescued cells. Supplement figure 10. The average body weight of mice after treated with TSN in LEG73 and LEG106. [file 13046_2023_2666_MOESM3_ESM.pdf]

Supplementary figures

Cancer type	Case number	Gender	Age	Pathological grading	T	N	M	Basic diseases
ESCC	LEG73	Male	74	IIIc	4a	1	0	Chronic bronchitis
ESCC	LEG106	Male	68	IIa	2	0	0	Hypertension
ESCC	LEG107	Female	64	IIIb	3	1	0	No
ESCC	LEG244	Male	66	IIb	3	0	0	Diabetes, bronchitis
ESCC	LEG367	Female	59	IIb	3	0	0	Breast cancer, bronchitis

Supplement figure 1. the clinical information of ESCC PDX cases.

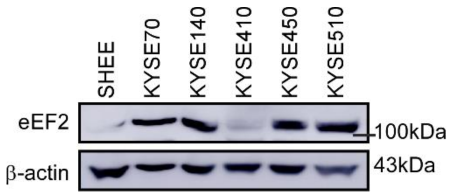

Supplement figure 2. eEF2 protein levels in different ESCC cell lines.

A

Gene	group	LPs/mL(AVG; 10^9)	SDV (10^9)
eEF2	Mock	4.48	0.13
	sh2	5.25	0.49
	sh3	4.93	0.23
TOP1	Mock	0.363	0.01
	sh3	0.976	0.20
	sh4	1.05	0.27
TOP2	Mock	0.58	0.02
	sh1	0.991	0.23
	sh4	1.28	0.49

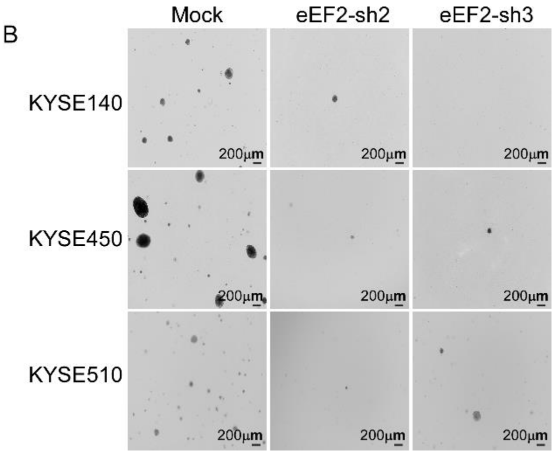

Supplement figure 3. A The concentration of shRNA virus used in this study. B The representative colony pictures of KYSE140, KYSE450 and KYSE510 after knock down of eEF2.

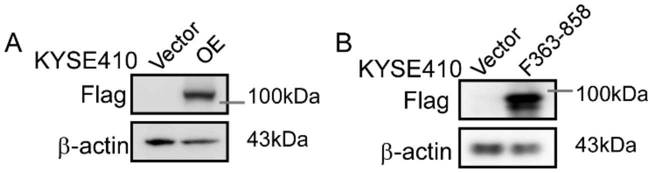

Supplement figure 4. Protein expression level in KYSE410. A Western blotting explored the protein expression level in KYSE410 after transfected with eEF2. B. The protein expression level in KYSE410 after transfected with F363-858 fragment.

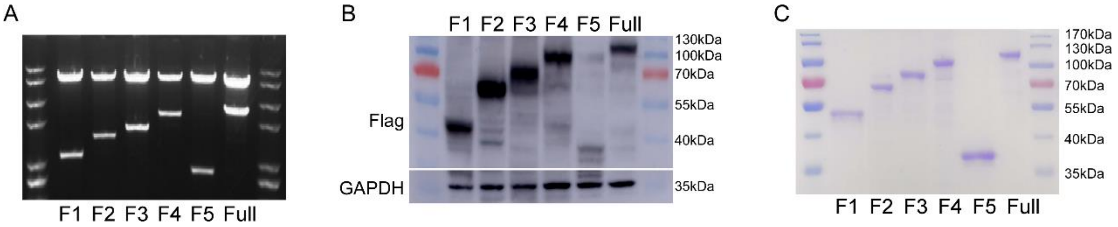

Supplement figure 5. The processes of eEF2 construction and purification. A The identification of different clone of fragments by restriction endonuclease. B The protein identification of different fragments by Western blot. C The purification of different fragments checked by comas blue staining.

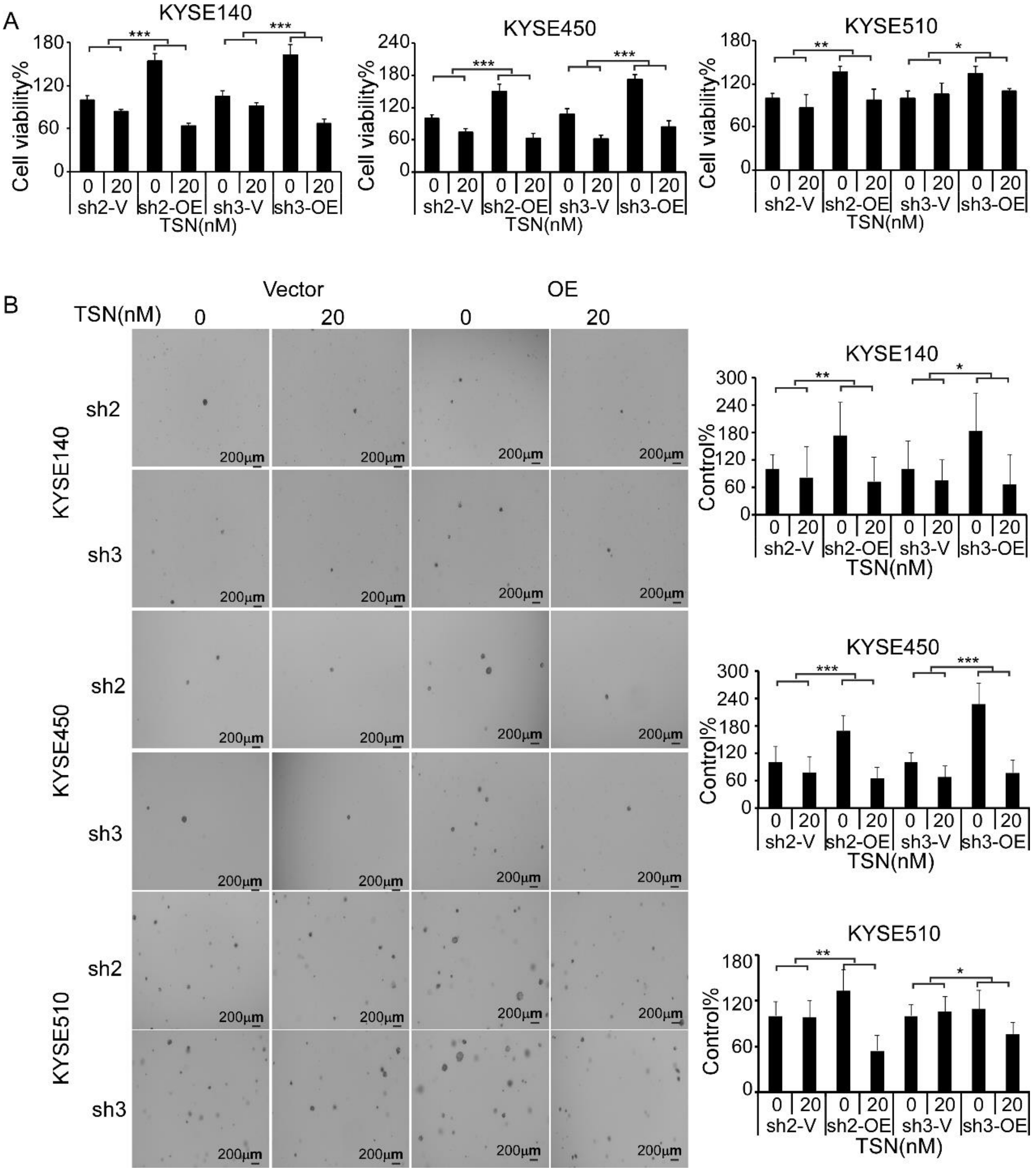

Supplement figure 6. Overexpressing eEF2 in eEF2 knockdown cells rescued the inhibitory effects of TSN on ESCC. A The cell viability of KYSE140, KYSE450 and KYSE510 after overexpressing eEF2 and treated by TSN. B The colony number of KYSE140, KYSE450 and KYSE510 after overexpressing

eEF2 and treated by TSN.

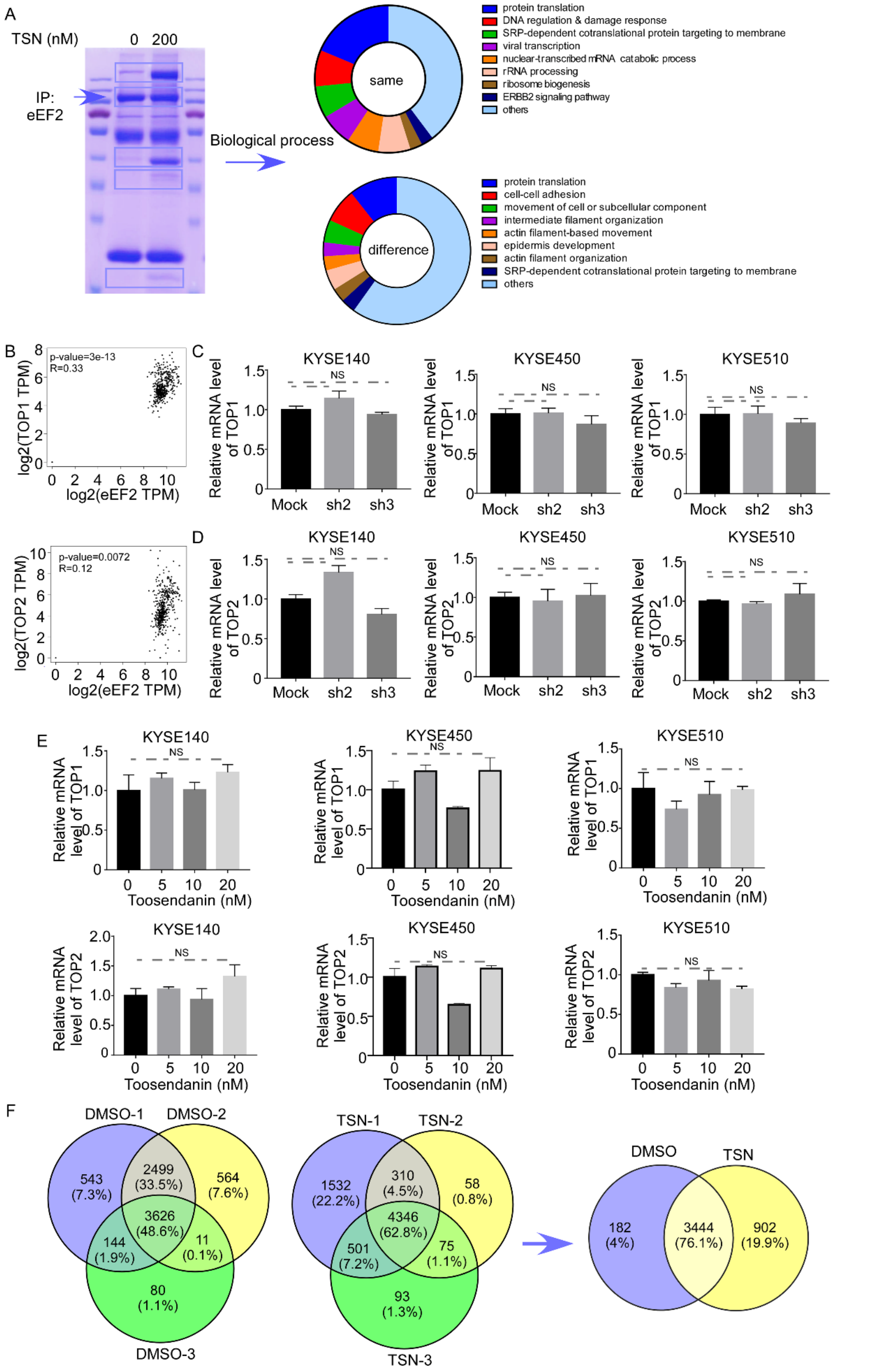

Supplement figure 7. TSN didn't affect the transcription of TOP1 and TOP2. A The different gel bands

were cut after co-incubation eEF2 antibody with or without TSN and subsequently analyzed the identified protein category according to cell biological process. B TOP1 and TOP2 expression correlation with eEF2 based on the GEPIA2 database. C&D The mRNA level of TOP1 and TOP2 in KYSE140, KYSE450 and KYSE510 after knocking down of eEF2. E The mRNA level of TOP1 and TOP2 in KYSE140, KYSE450 and KYSE510 after treated by TSN. F RIP-Seq results when conducted with or without the existence of TSN. Each group has three replicates. After obtained the merged mRNA in DMSO and TSN treated group, the merged mRNAs were compared between DMSO and TSN group.

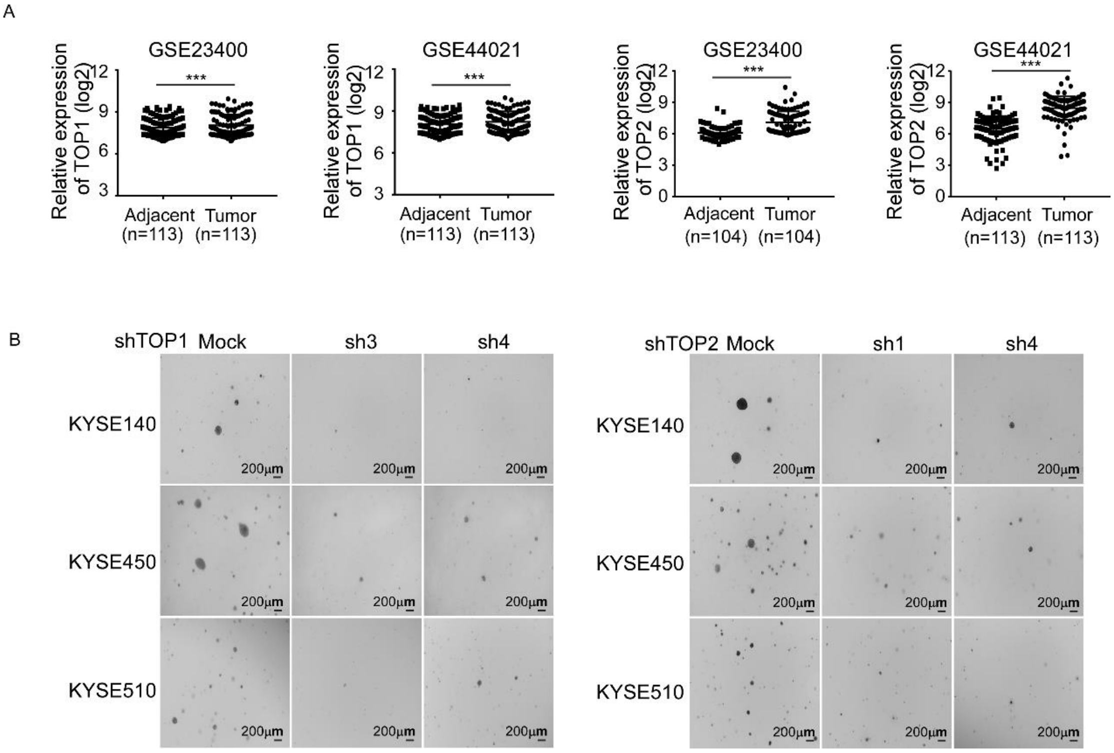

Supplement figure 8. TOP1 and TOP2 expression level and colony pictures. A Expression levels of TOP1 and TOP2 in gene microarray of ESCC. B The representative colony pictures of KYSE140, KYSE450 and KYSE510 after knock down of TOP1 and TOP2.

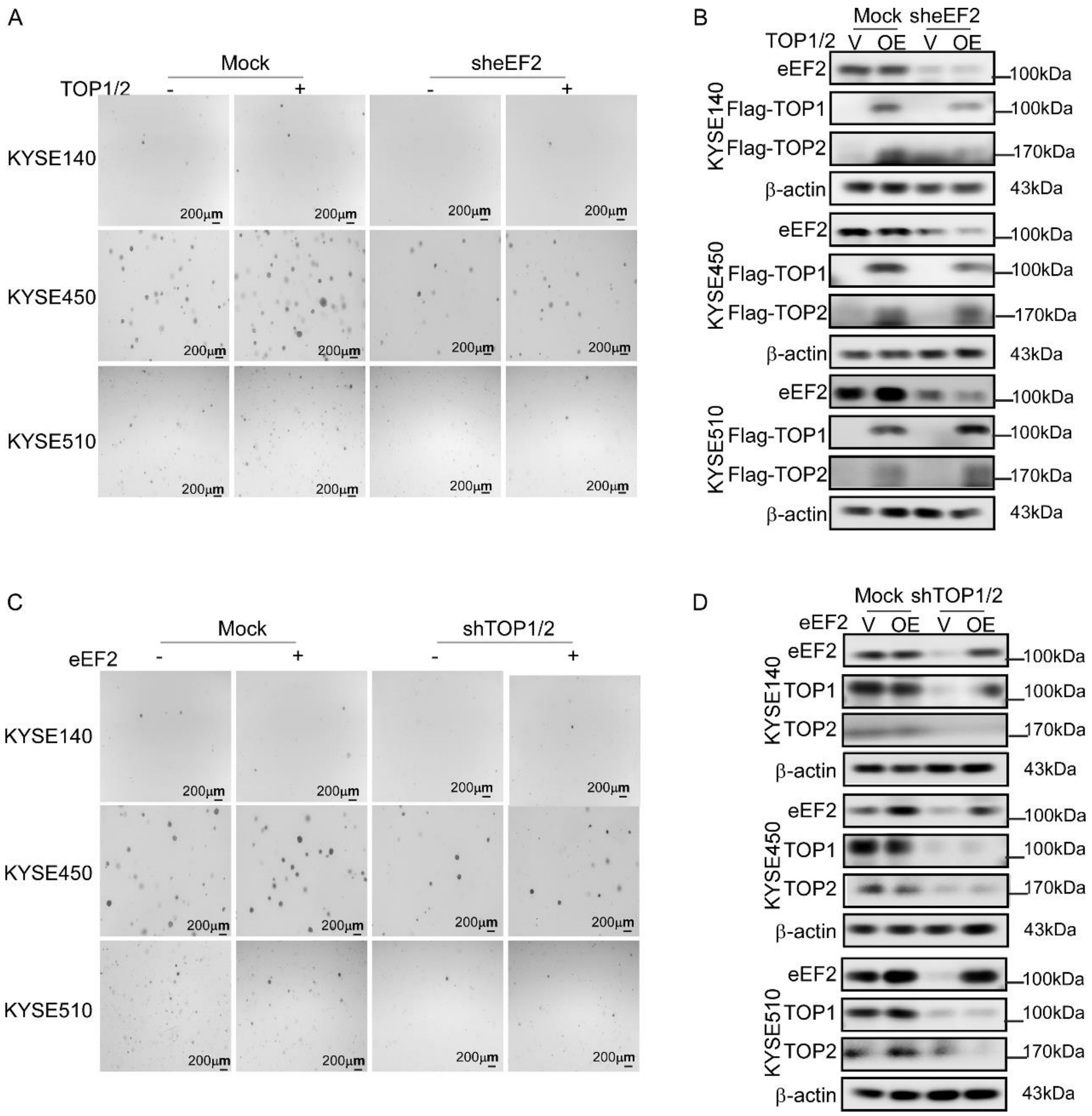

Supplement figure 9. Representative colony pictures in KYSE140, KYSE450 and KYSE510 rescued cells. A Representative colony pictures of eEF2 knocking down, TOP1 and TOP2 overexpressed cells. B Expression levels of eEF2, TOP1 and TOP2 in eEF2 knocking down, TOP1 and TOP2 overexpressed cells. C Representative colony pictures of TOP1 and TOP2 knocking down, eEF2 overexpressed cells. D Expression levels of eEF2, TOP1 and TOP2 in TOP1 and TOP2 knocking down, eEF2 overexpressed cells.

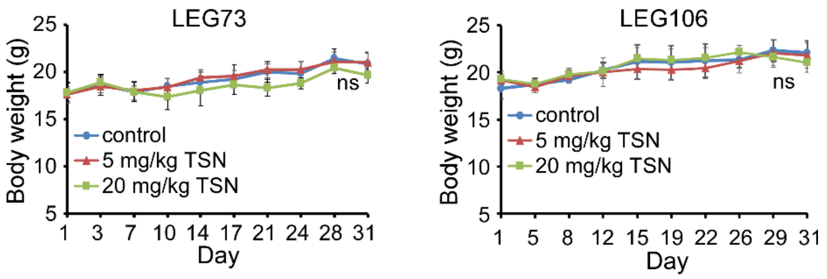

Supplement figure 10. The average body weight of mice after treated with TSN in LEG73 and LEG106.
